# The Interrelations Between Social Class, Personal Relative Deprivation, and Prosociality

**DOI:** 10.1177/1948550616673877

**Published:** 2016-11-11

**Authors:** Mitchell J. Callan, Hyunji Kim, Ana I. Gheorghiu, William J. Matthews

**Affiliations:** 1 Department of Psychology, University of Essex, Colchester, United Kingdom; 2 Department of Psychology, University of Cambridge, Cambridge, United Kingdom

**Keywords:** socioeconomic status, personal relative deprivation, social class, prosocial beliefs, prosocial behavior

## Abstract

We propose that personal relative deprivation (PRD)—the belief that one is worse off than similar others—plays a key role in the link between social class and prosociality. Across multiple samples and measures (total *N* = 2,233), people higher in PRD were less inclined to help others. When considered in isolation, neither objective nor subjective socioeconomic status (SES) was meaningfully associated with prosociality. However, because people who believe themselves to be at the top of the socioeconomic hierarchy are typically low in PRD, these variables act as mutual suppressors—the predictive validity of both is enhanced when they are considered simultaneously, revealing that both higher subjective SES and higher PRD are associated with lower prosociality. These results cast new light on the complex connections between relative social status and people’s willingness to act for the benefit of others.

There has recently been intense scholarly and popular interest in the effects of social class on people’s beliefs, behavior, and everyday functioning (e.g., Hooker, 2015; Kraus, Piff, Mendoza-Denton, Rheinschmidt, & Keltner, 2012). Of particular interest is the effect of social class on prosocial behavior, with research suggesting that people higher in objective and/or subjective socioeconomic status (SES) are greedier and less prosocial than people lower in SES (e.g., Piff, Kraus, Côté, Cheng, & Kelter, 2010), perhaps because those at the top of the social ladder are free to pursue individual goals while those at the bottom must use communalistic, attachment-related behaviors to deal with the increased threats and hostility that they face (Kraus et al., 2012). This negative relationship between SES and helping others has been found with diverse measures of prosociality, including self-reported attitudes and behavior in economic games (Piff et al., 2010). Similarly, it has been found using conventional indicators of SES (e.g., income and education; Stellar, Manzo, Kraus, & Keltner, 2012) and using people’s self-perceived rank in the national population (subjective socioeconomic status [SSS]; e.g., Piff, Stancato, Côté, Mendoza-Denton, & Keltner, 2012).

Objective SES and SSS are, however, likely not the only indicators of social status relevant to people’s willingness to act for the benefit of others. We sought a new perspective by investigating the contribution of *personal relative deprivation* (PRD) to the relationship between social class and prosociality. PRD refers to resentment stemming from the belief that one is deprived of desired and deserved outcomes compared to some referent target (Crosby, 1976; Smith, Pettigrew, Pippin, & Bialosiewicz, 2012). Like social class, PRD pertains to an individual’s relative status within a socioeconomic hierarchy. However, rather than emphasizing status within a national population, PRD reflects more local, specific, interpersonal comparisons such as those we make with “similar others” (e.g., friends, coworkers) because such comparisons provide the most diagnostic information for self-evaluation (Wood, 1989). In addition, PRD concerns the feelings of resentment and unfairness that may result from these comparisons rather than subjective or objective status per se. For example, Callan, Ellard, Shead, and Hodgins (2008) found that participants who were told that they had less (vs. the same) discretionary income than their peers reported greater perceived unfairness and resentment, even though actual socioeconomic position was fixed across experimental conditions.

Correspondingly, there is only a moderate negative correlation between PRD and objective SES or SSS (Callan, Kim, & Matthews, 2015). For example, a lawyer might be well educated, hold a prestigious position in a fancy law firm, and make a lot of money but nonetheless experience a sense of unfairness and resentment when she thinks about what she has compared to other lawyers in her firm, whereas a low-paid retail clerk may feel less deprived than his unemployed former classmates. Thus, while people at the top of the SES ladder will typically experience less resentment about their lot in life than those at the bottom, the relationship is far from perfect, raising the possibility that PRD contributes to prosociality over and above social class.

Although PRD and social class are correlated, theoretical predictions for their respective associations with prosociality are different—people higher in objective SES and/or SSS are expected to be more solipsistic and therefore less prosocial (Kraus et al., 2012), whereas a long tradition of research into the psychology of justice suggests that people higher in PRD (or lower in perceived status relative to similar others) should be less prosocial because they feel their own personal deservingness concerns are not being met (Callan, Ellard, Shead, & Hodgins, 2008). For example, recounting past personal injustices increases selfish behaviors (Zitek, Jordan, Monin, & Leach, 2010), pay inequity in workplace contexts reduces helping and increases dishonest behaviors (Cohen-Charash & Spector, 2001; John, Loewenstein, & Rick, 2014; Miller, 1977), and believing that the world does not treat one fairly correlates with reduced charitable giving (Bègue, 2014; Bègue, Charmoillaux, Cochet, Cury, & De Suremain, 2008).

Consistent with this analysis, Zhang, Liu, and Tian (2016) recently found that self-reported PRD was associated with weaker prosocial beliefs and behaviors, although these authors did not examine the interrelations among PRD, social class, and prosociality. In fact, the contributions of these indicators of social status to prosociality have previously only been studied in isolation. However, modeling these variables simultaneously may illuminate their respective contributions to prosociality: If PRD and social class are both negatively related to prosociality but also negatively related to one another, then modeling both variables simultaneously will improve the predictive validity of each of them. This pattern is indicative of mutual statistical suppression, which occurs when the magnitudes of regression coefficients are larger when modeled together than when they are modeled alone (for descriptions of the various types of statistical suppression effects, see Cohen & Cohen, 1975; Conger, 1974; Paulhus, Robins, Trzesniewski, & Tracy, 2004; Tzelgov & Henik, 1991). Such suppression effects occur because criterion-irrelevant variance shared between predictors is partialled out or suppressed, thereby strengthening the relationships between the predictors and the criterion.

Although often considered statistical nuisances or artifacts, statistical suppression effects can be replicable and of substantive interest. For example, Paulhus, Robins, Trzesniewski, and Tracy (2004) found that measures of shame and guilt were mutual suppressors of self-reported aggression. Although shame and guilt are moderately positively correlated, they produce divergent outcomes for aggression—shame is positively correlated with aggression, whereas guilt is negatively correlated with aggression. Paulhus et al. (2004) found that modeling shame and guilt together, thereby removing their shared variance, significantly enhanced their opposing associations with aggression. In a similar vein, the negative relationship between social class and prosocial behavior may be strengthened once we control for PRD (and vice versa). That is, the full predictive power of PRD and social class for prosociality might be revealed only when criterion-irrelevant variance shared between them is removed.

We report five studies in which we measured individual differences in PRD, objective SES *and* SSS, and various individual difference measures of prosocial beliefs and behaviors. Most previous studies investigating the association between social class and prosociality have considered objective SES and SSS as interchangeable indicators of social class (Kraus et al., 2012). Because research in other domains suggests that SSS sometimes better predicts outcomes of interest than does objective SES (e.g., mental health; Callan, Kim, et al., 2015), we included both variables in all studies to explore their potentially unique associations with prosociality.

Our goals were (1) to test whether the relationship between social class and prosociality replicates across highly powered studies that use other samples, procedures, and measures (cf. Funder et al., 2014; Open Science Collaboration, 2015; Pashler & Wagenmakers, 2012); (2) to clarify the relative contributions of objective SES and SSS to this relationship; (3) to determine whether PRD predicts prosociality over and above the effects of social class; and (4) to examine whether PRD and social class suppress each others’ contributions to prosociality, such that social class and PRD enjoy greater predictive power when they are modeled simultaneously.

## Method

### Sampling

The minimum required sample sizes were fixed ahead of data collection to obtain at least 80% power to detect bivariate correlations of *r* = .15 (i.e., small-to-medium effect sizes; two tailed, α = .05). The final sample sizes were not precisely predetermined because some participants were excluded from analysis (see below). We report all measures we employed across studies.

### Participants

A total of 2,233 participants across five studies were recruited either through Amazon’s Mechanical Turk (Studies 1–3 and 5) or Prolific.ac (Study 4). Sample characteristics for each study are shown in Table 1. An additional 173 participants were excluded from analyses for incorrectly answering an attention or comprehension check item (e.g., “Attention check. Please select ‘strongly disagree’”; *n* = 119) or, to ensure independence of data, having duplicate Internet protocol (IP) addresses between or within studies (*n* = 54; we retained the data for only the first occurrence of each IP). An additional five participants from Study 4 were removed because they reported not living in the United Kingdom but were asked to report their income in pound sterling.

**Table 1.** Sample Characteristics.

Characteristics	Study 1 (*n* = 564)	Study 2 (*n* = 392)	Study 3 (*n* = 546)	Study 4 (*n* = 338)	Study 5 (*n* = 393)
*M* age (*SD*)	34.36 (10.84)	32.60 (10.90)	33.68 (11.42)	31.18 (10.33)	33.72 (11.02)
Gender (%)					
Male	51.2	56.4	51.8	38.2	57.8
Female	48.8	42.9	48.0	61.8	42.2
Unreported	—	0.8	0.2	—	—
*M* Annual household income (*SD*)	53.4 k (US$) (36.6 k)	54.4 k (US$) (41.6 k)	52.3 k (US$) (41.8 k)	33.9 k (£) (27.8 k)	51.9 k (US$) (36.7 k)
Education (%)					
Did not finish high school	0.5	0.3	0.9	*M* = 5.01^a^ (*SD* = 2.93)	1.3
High school graduation	41.8	43.1	42.7		34.1
College graduation	43.3	47.2	46.2		53.2
Postgraduate degree	14.4	9.4	10.3		11.5
Ethnicity (%)					
White/Caucasian	79.1	73.5	80.0	—	75.8
African American	7.1	6.6	6.2	—	6.1
Hispanic	5.3	5.4	4.9	—	4.6
Asian	6.2	12.5	6.4	—	8.7
Native American	0.4	0.3	0.7	—	1.3
Pacific Islander	—	—	0.2	—	0.8
Other	2.0	1.8	1.5	—	2.8

^a^Number of years of formal education since the age of 16 years old.

### Measures and Procedures

We measured participants’ PRD, SSS, and objective SES in the same way across studies. The primary differences between studies were the measures we used to assess prosociality (described below). We used Callan, Shead, and Olson’s (2011) 5-item Personal Relative Deprivation Scale (PRDS). The PRDS gauges people’s feelings and beliefs associated with comparing their outcomes with the outcomes of similar others (“I feel deprived when I think about what I have compared to what other people like me have”; “I feel privileged compared to other people like me” (reversed); “I feel resentful when I see how prosperous other people like me seem to be”; When I compare what I have with what others like me have, I realize that I am quite well off” (reversed); “I feel dissatisfied with what I have compared to what other people like me have”). The PRDS has acceptable reliability (Callan, Kim, et al., 2015) and has been shown to predict a variety of theoretically relevant consequences of higher PRD, such as increased gambling urges (Callan, Shead, & Olson, 2015), greater temporal discounting (Callan, Shead, & Olson, 2011; Mishra & Novakowski, 2016), and worse health (Callan, Kim, et al., 2015; Mishra & Carleton, 2015). Participants responded to the items using a 6-point scale (1 = *strongly disagree* to 6 = *strongly agree*); higher values indicate higher PRD.

For our measure of SSS, participants completed MacArthur’s Scale of Subjective Social Status (Adler, Epel, Castellazzo, & Ickovics, 2000). We presented participants with an image of a 10-rung ladder representing “where people stand in the United States (United Kingdom),” with the top (bottom) rung representing people are the best (worst) off in terms of education, income, and occupational status. Participants indicated where they stood at that time in their lives by clicking on a ladder rung within the image. Higher scores indicate higher SSS. Due to technical problems, 36 participants across studies did not provide a response for the SSS measure. We replaced these missing values with predicted scores from regression analyses including participants’ PRDS, household income, and education as predictors of SSS. Removing these participants listwise yielded virtually identical results.

Following Piff, Kraus, Côté, Cheng, and Kelter (2010, Study 3), our measure of objective SES was a composite of participants’ self-reported annual household income before taxes and their educational attainment. For Studies 1–3 and 5, participants indicated their income by choosing one of eight categories (1 = *less than US$15,000* to 8 = *greater than US$150,000*). For Study 4, participants indicated their income among 18 income categories (1 = *less than £5,000* to 18 = *£85,001 and above*). Across studies, income responses were converted into estimates of absolute income using the category midpoints, adopting Parker and Fenwick’s (1983) median-based Pareto curve estimator for the highest, open-ended category (Matthews, Gheorghiu, & Callan, 2016).^1^ For our U.S. samples, participants indicated their highest level of educational attainment among four options (1 = *did not finish high school*, 2 = *high school graduation*, 3 = *college graduation*, 4 = *postgraduate degree*). For the UK sample, participants reported the number of years of formal education they achieved since the age of 16. For each study, income responses and educational attainment were standardized and summed to form composite measures of objective SES (cf. Piff et al., 2010). Because researchers interested in the effects of social class have operationalized objective SES in a variety of ways (e.g., sometimes income alone, education alone, composites of income, and education; see Kraus et al., 2012), we also report the correlations for income and education separately, but we draw our main conclusions from the composites of income and education. The study-specific measures and procedures were as follows.

### Study 1: Social Value Orientation (SVO)

Participants completed Van Lange, Agnew, Harinck, and Steemers’ (1997) widely used and valid (Balliet, Parks, & Joireman, 2009) measure of SVO. Participants imagined that they were paired with another person and, across nine decomposed games, had to choose one of three combinations of points to give to themselves and to the other person. For each choice, one option represented a prosocial, egalitarian orientation (e.g., you both get 500 points), while the other options represented individualistic (e.g., you get 580, the other gets 320) or competitive orientations (e.g., you get 500, the other gets 100). Our measure of prosociality was the number of egalitarian choices participants made across the nine decomposed games (cf. Piff et al., 2010), with higher scores representing greater prosociality. In Study 1, we presented the PRDS, SSS ladder, and SVO measure in a counterbalanced order between participants. Next, participants reported their age, gender, ethnicity (among seven options; see Table 1), household income, and educational attainment.

### Study 2: Community Aspirations

We used the aspiration index (AI; Kasser & Ryan, 1996) to assess the importance participants placed on contributing to “community.” The version of the AI we used contained seven different life goal categories (affiliation, attractive appearance, community feeling, physical fitness, financial success, social recognition, and self-acceptance), and each category was assessed with 4 or 5 items (32 items total). Participants rated the importance of each statement to them on a scale ranging from 1 (*not at all important*) to 5 (*very important*). We operationalized prosociality in Study 2 as the importance participants placed on community as a future goal in their lives (5-items, e.g., “You will donate time or money to charity”; “You will help people in need”) relative to the perceived importance of other life goals. Following standard scoring procedures for the AI (e.g., Sheldon, Sheldon, & Osbaldiston, 2000), this was achieved by subtracting each participant’s mean across the full AI from the mean of the 5 items assessing their perceived importance of community feelings. Higher scores indicate greater perceived importance of contributing to community relative to other life goals. We administered the PRDS, SSS ladder, and AI in a counterbalanced order across participants. Next, participants reported their age, gender, ethnicity, household income, and educational attainment.

### Study 3: Dictator Game

Participants played an incentivized dictator game where they indicated how they would distribute US$10 between themselves and the next participant (see Supplemental Material for the instructions). At the end of the study, we paid bonuses to 10 randomly selected “dictators” according to how much of the US$10 they said they would keep for themselves. We paid another 10 randomly selected participants according to how much these dictators said they would give to the next participant (participants were informed of this in advance). Participants completed the PRDS, SSS, and objective SES measures (in a counterbalanced order between participants) before completing the dictator game and providing demographic information per Study 1.

### Study 4: Communal Orientation I

Participants completed Clark, Oullette, Powell, and Milberg’s (1987) 14-item Communal Orientation Scale (COS; e.g., “I believe people should go out of their way to be helpful”; “I’m not the sort of person who often comes to the aid of others”). The items were rated using a scale ranging from 1 (*extremely uncharacteristic of me*) to 7 (*extremely characteristic of me*). The COS was designed to assess individual differences in the extent to which people believe that people should help and care for others and has been shown to predict actual helping behavior (Clark, Oullette, Powell, & Milberg, 1987).

Following common use of the COS (e.g., Piff, Stancato, Martinez, Kraus, & Keltner, 2012), our original analysis strategy was to use the full scale. However, given Clark et al. (1987) provided evidence for more than one factor for the COS, we conducted a principal component analysis (PCA) of our data. This analysis revealed two distinct components—one that measures people’s desires to *help others* (10 items; e.g., I believe people should go out of their way to be helpful) and another that measures people’s desires to *receive help from others* (4 items; e.g., “When I have a need, I turn to others I know for help”). Given that our primary interest was people’s prosociality (i.e., beliefs about helping others), we focused on the *help others* subscale in our analysis and discussion. The results of the PCA and the correlations among our focal predictors and the full COS and its two subscales are reported in the Supplemental Materials. In addition, one impetus for Study 5 was to confirm this two-component structure of the COS with a confirmatory factor analysis (which showed that the two factor solution better fitted the data than the one factor solution; see Supplemental Materials).

Our second interest in Study 4 was to explore the role that beliefs about deservingness play in the relation between PRD and people’s desires to help others. To this end, participants also completed Lipkus, Dalbert, and Siegler’s (1996) Belief in a Just World for the Self (BJW-S; e.g., “I feel that I get what I deserve”) and Belief in a Just World for Others (BJW-O; “I feel that people get what they deserve”) scales. Research has shown that the BJW-S tends to correlate positively, and the BJW-O negatively, with prosocial beliefs and behavior (Bègue et al., 2008).

Participants completed the PRDS and SSS (in a counterbalanced order) before completing the COS and BJW scales and then provided their age, gender, income, and education (ethnicity was not measured in Study 4).

### Study 5: Communal Orientation II

Study 5 used the same measures and procedure as Study 4 except that instead of including the BJW scales, we included 2 items that more specifically gauge people’s beliefs about not getting what they feel they deserve relative to similar others (“When I think about what I have compared to what other people like me have, I feel like I am getting less than I deserve”; “I think it’s unfair how well off other people like me seem to be”). These items appeared at the end of the PRDS, were rated using the same 6-point scale, and were averaged to form one composite measure of perceived unfairness (*r* = .66 between items, *p* < .001).

## Results

Data for all studies are available at https://osf.io/h24zj/. Table 2 shows the correlations among PRD, SSS, objective SES, and the prosociality measures for each study. Consistent with Callan, Kim, and Matthews’s (2015) findings, PRD and SSS correlated negatively across studies: People with higher SSS experience less PRD. With the exception of Study 4, PRD correlated significantly with the prosocial measures across studies, such that higher PRD was associated with lower egalitarian SVOs, less giving during the dictator game, lower relative importance of contributing to the community, and a lower desire to help others. For the most part, SSS and objective SES did not correlate significantly with the prosociality measures. Analysis of the data standardized within studies and collated across studies (*N* = 2,233; see Table 3) showed that PRD significantly correlated with prosociality, whereas SSS and objective SES did not.

**Table 2.** Descriptive Statistics and Intercorrelations for Measures Used Across Studies.

Measures	Mean (*SD*)	1	2	3	3a	3b	4
Study 1 (*n* = 564)							
1. PRD	3.15 (1.01)	(.83)					
2. SSS	4.87 (1.74)	−.421*	—				
3. Objective SES	—	−.188*	.508*	—			
3a. Income	53.4 k (US$) (36.6 k)	−.285*	.556*	.777*	—		
3b. Education	2.71 (0.71)	−.008	.234*	.777*	.207*	—	
4. SVO	5.42 (4.05)	−.117*	−.015	.001	−.006	.007	—
Study 2 (*n* = 392)							
1. PRD	3.11 (1.10)	(.85)					
2. SSS	4.79 (1.77)	−.506*	−				
3. Objective SES	—	−.328*	.569*	—			
3a. Income	54.4 k (US$) (41.6 k)	−.298*	.527*	.754*	—		
3b. Education	2.66 (0.65)	−.197*	.332*	.754*	.138*	—	
4. AI community	0.12 (0.73)	−.176*	−.126^†^	−.068	−.108^†^	.005	—
Study 3 (*n* = 546)							
1. PRD	3.07 (1.04)	(.86)					
2. SSS	4.84 (1.73)	−.433*	—				
3. Objective SES	—	−.247*	.514*	—			
3a. Income	52.3 k (US$) (41.8 k)	−.222*	.505*	.794*	—		
3b. Education	2.66 (0.67)	−.170*	.311*	.794*	.261*	—	
4. DG	3.42 (2.19)	−.126*	−.024	.028	−.024	.068	—
Study 4 (*n* = 338)							
1. PRD	3.00 (0.95)	(.79)					
2. SSS	5.27 (1.68)	−.432*	—				
3. Objective SES	—	−.179*	.410*	—			
3a. Income	33.9 k (£) (27.8 k)	−.180*	.325*	.700*	—		
3b. Education	5.01^a^ (2.93)	−.069	.249*	.700*	−.021	—	
4. COS give help	5.22 (0.93)	−.099	−.092	−.165*	−.132^†^	−.099	—
Study 5 (*n* = 393)							
1. PRD	3.15 (1.00)	(.81)					
2. SSS	4.94 (1.74)	−.527*	—				
3. Objective SES	—	−.299*	.533*	—			
3a. Income	51.9 k (US$) (36.7 k)	−.293*	.528*	.794*	—		
3b. Education	2.75 (0.67)	−.181*	.318*	.794*	.262*	—	
4. COS give help	5.11 (1.08)	−.238*	.041	.004	.068	−.062	—

*Note*. α Reliabilities are presented in parentheses along the diagonals where applicable. PRD = personal relative deprivation; SSS = subjective socioeconomic status; Objective SES = composite of income and education; SVO = social value orientation; AI community = relative importance of community as a life goal; DG = dictator game; COS = Communal Orientation Scale.

^a^Number of years of formal education since the age of 16.

*p < .01. †*p* < .05.

**Table 3.** Correlations Among Measures for the Standardized and Collated Data.

Measures	1	2	3	3a	3b	4
1. PRD	—					
2. SSS	−.459*	—				
3. Objective SES	−.246*	.511*	—			
3a. Income	−.257*	.499*	.769*	—		
3b. Education	−.121*	.287*	.769*	.183*	—	
4. Prosociality	−.148*	−.038	−.027	−.034	−.007	—

*Note*. PRD = personal relative deprivation; SSS = subjective socioeconomic status; SES = socioeconomic status; Objective SES = composite of income and education.

*p < .01.

### Suppression Analyses

PRD correlated negatively with prosociality, and both SSS and objective SES generally correlated negatively with prosociality (although not significantly), yet PRD and SSS and PRD and objective SES correlated *negatively* with each other. This pattern suggests the possibility of mutual suppression. To test for suppressor effects, we regressed the prosociality measures onto PRD, SSS, and objective SES alone and then in combination with the other predictors for each study and with the data collated across studies (all variables were standardized before conducting analyses). For each study and with the data combined across studies, the predictive validity of PRD and SSS were enhanced when PRD, SSS, and objective SES were modeled together compared to when they were modeled alone (see “Total Suppression Effect” column, with positive values indicating increased predictive validity). We complemented these analyses with regression commonality analysis (Nimon & Reio, 2011), which is a method of variance partitioning that is well suited to isolating predictors that are involved in suppressor situations (see online Supplemental Materials).

Because suppressor effects are a special case of indirect effects (MacKinnon, Krull, & Lockwood, 2000), we tested the significance of these suppressor effects (i.e., whether there is a significant increase in a predictor’s regression weight when modeled with the other predictors compared to when it is modeled alone) using Preacher and Hayes’s (2008) bootstrapping procedure (10,000 resamples for each analysis). Shown in Table 4, the regression weight for PRD was significantly increased in three of five studies, SSS in four of five studies, and objective SES in zero of five studies (based on the 95% bias-corrected and accelerated confidence intervals [95% BCa CI] crossing zero or not). Analyses performed on the collated data^2^ demonstrated that PRD and SSS were significant suppressors, while the relation between objective SES and prosociality did not change significantly from its initial validity.

**Table 4.** Multiple Regression Results for Studies 1–5 and the Collated Data.

Criterion Predictors	*R* ^2^	β_alone_ (*p*) [95% CI]	β_with other predictors_ (*p*) [95% CI]	Total Suppression Effect	95% BCa CI Suppression Effect
SVO	.019				
PRD		−.117 (.005) [−.199, −.035]	−.150 (.001) [−.241, −.060]	.033	[−.004, .073]
SSS		−.015 (.728) [−.098, .068]	−.086 (.102) [−.189, .017]	.071	[.008, .133]
Objective SES		.001 (.986) [−.082, .084]	.016 (.739) [−.079, .112]	−.016	[−.065, .034]
AI community	.093				
PRD		−.176 (<.001) [−.274, −.078]	−.323 (<.001) [−.433, −.213]	.147	[.081, .226]
SSS		−.126 (.013) [−.224, −.027]	−.280 (<.001) [−.407, −.154]	.155	[.072, .246]
Objective SES		−.068 (.176) [−.168, .031]	−.015 (.804) [−.131, .101]	−.054	[−.131, .019]
Dictator Game	.025				
PRD		−.126 (.003) [−.209, −.042]	−.166 (<.001) [−.258, −.073]	.040	[.002, .082]
SSS		−.024 (.578) [−.108, .060]	−.120 (.024) [−.225, −.016]	.097	[.033, .162]
Objective SES		.028 (.521) [−.057, .112]	.048 (.328) [−.049, .146]	−.021	[−.076, .032]
COS give help (United Kingdom)	.052				
PRD		−.099 (.068) [−.206, .007]	−.171 (.004) [−.287, −.055]	.072	[.018, .133]
SSS		−.092 (.092) [−.199, .015]	−.103 (.108) [−.228, .023]	.011	[−.066, .086]
Objective SES		−.165 (.002) [−.271, .059]	−.154 (.009) [−.269, −.039]	−.012	[−.063, .040]
COS give help (United States)	.067				
PRD		−.238 (<.001) [−.334, −.141]	−.300 (<.001) [−.413, −.187]	.062	[−.003, .129]
SSS		.041 (.419) [−.058, .140]	−.100 (.126) [−.228, .028]	.141	[.050, .234]
Objective SES		.004 (.939) [−.096, .103]	−.033 (.574) [−.146, .081]	.036	[−.028, .108]
Collated (*N* = 2,233)	.037				
PRD		−.148 (<.001) [−.189, −.107]	−.210 (<.001) [−.257, −.165]	.062	[.041, .085]
SSS		−.038 (.072) [−.080, .003]	−.128 (<.001) [−.180,−.077]	.090	[.058, .123]
Objective SES		−.027 (.208) [−.068, .015]	−.013 (.595) [−.061, .034]	−.013	[−.039, .013]

*Note*. PRD = personal relative deprivation; SSS = subjective socioeconomic status; Objective SES = composite of income and education; SVO = social value orientation; β_alone_ = zero-order correlation between predictor and criterion; Total suppression effect = the change in β from β_alone_ when modeled with the other two predictors; 95% BCa CI suppression effect = 95% bias-corrected and accelerated confidence intervals for the total suppression effect.

### Is the Relationship Between PRD and Prosociality Mediated by Beliefs About Fairness?

Studies 4 and 5 included measures to gauge participants’ beliefs about not getting what they personally deserve in general (BJW-S; Study 4) or relative to people like them (Study 5). Our primary interest was whether PRD correlates negatively with people’s self-reported interest in helping others partly through beliefs about personal deservingness (while controlling for the BJW-O; cf. Khera, Harvey, & Callan, 2014). Although PRD correlated significantly with BJW-S in the expected direction in Study 4 (*r* = −.48, *p* < .001; while controlling for BJW-O, β = −.43, *p* < .001), BJW-S did not correlate significantly with participants’ self-reported desires to help others (*r* = −.085, *p* = .12; while controlling for BJW-O, β = .019, *p* = .76). Consistent with previous research (Bègue et al., 2008), BJW-O was negatively associated with helping others (*r* = −.205, *p* < .001; while controlling for BJW-S, β = −.214, *p* < .001).

In Study 5, PRD was significantly related to the participants’ perceived unfairness of what they have compared to what similar others have (*r* = .759, *p* < .001) and perceived unfairness correlated significantly with the desire to help others (*r* = −.265, *p* < .001). Bootstrapped mediation analyses showed that perceived unfairness mediated the relation between PRD and the desire to help others while controlling for SSS and objective SES (10,000 resamples; unstandardized total effect = −.323; indirect effect = −.161, 95% BCa CI of [−0.30 and −0.026]). It is important to note that although these latter findings are consistent with our theoretical analysis, the high correlation between PRD and perceived unfairness and the cross-sectional nature of our data prevents us from making strong claims about causal direction (but see Callan et al., 2008, for experimental evidence showing that adverse social comparisons affect perceived unfairness).

### Does Social Class Moderate the Relation Between PRD and Prosociality?

Our theoretical perspective suggests that even people who are high in social class can feel resentful about what they have compared with what others like them have and that these feelings reduce prosocial tendencies. An alternative possibility, however, is that the relation between PRD and prosociality is moderated by social class, such that the relation between PRD and prosociality might occur only for people particularly low in social class. However, neither objective SES, *b* = .003, *t*(2,228) = .135, *p* = .893 (adjusting for SSS), nor SSS, *b* = −.021, *t*(2,228) = −1.073, *p* = .283 (adjusting for objective SES), significantly moderated the relation between PRD and prosociality across the collated data. Thus, being high in social class does not appear to insulate people from the potentially negative consequences of PRD for prosociality.

## General Discussion

Relative social status is a complex construct encompassing, among other things, objective socioeconomic indicators (SES), a subjective assessment of one’s position in the national distribution (SSS), and the sense of whether one is getting what one deserves relative to similar others (PRD). Our results illustrate the distinct relationships between these components of social status and the willingness to act for the benefit of other people.

We found that prosociality was not significantly related to objective SES but was negatively related to both PRD and SSS when these variables were modeled together. More specifically, PRD and SSS were mutual suppressors: The effects of both predictors were strengthened when they were considered simultaneously. Indeed, when SSS was considered in isolation, it was not reliably related to prosociality.

The robust negative association between PRD and prosociality adds to a growing body of evidence that PRD is an important predictor of social outcomes, behaviors, and attitudes (Smith et al., 2012). Central to these effects are the feelings of resentment and unfairness that arise when people feel that similar others have more than they do. Prosocial behavior is often motivated by principles of reciprocity and sharing (Zhang & Epley, 2009) that lose relevance if people believe they are not getting what they deserve compared to others and when the goal is an immediate enhancement of one’s status that will help to redress this perceived unfairness (Callan et al., 2011). The negative relationship between PRD and prosociality can therefore be seen as part of a broader principle in which unfavorable social comparisons elicit feelings of unfairness and resentment that lead to a focus on short-term self-advancement.

This association between PRD and prosociality provides new insights into the relationship between social class and helping behavior. Previous studies of this relationship have produced mixed results: Social psychologists have found that higher social class corresponds to lower prosociality (e.g., Piff et al., 2010), but other researchers have sometimes found the opposite or no effect (Korndörfer, Egloff, & Schmukle, 2015; Van Doesum, Tybur, & Van Lange, 2017), leading to descriptions of the association as “fragile” (Korndörfer et al., 2015, p. 39), and the hunt for factors—including geographical region (e.g., Côté, House, & Willer, 2015) and whether making prosocial actions are public versus private (Kraus & Callaghan, 2016)—that might moderate the relationship.

Consistent with these difficulties in replicating the negative association between social class and prosociality found in previous studies, our data show that when considered in isolation, neither objective SES nor SSS showed a meaningful association with prosociality. However, when SSS and PRD are modeled simultaneously, the effects of both predictors strengthened, revealing a significant negative association between SSS and helping other people. Such suppression is rare in the behavioral sciences (e.g., Cohen & Cohen, 1975). In the present case, it arises because, although PRD and SSS are distinct constructs, there is a moderate negative correlation between them: A highly paid lawyer may resent the corner office of her coworker but, on average, affluent lawyers have less scope for unfavorable upward comparisons than people at the bottom of the distribution. This means that, while people of low SSS may, *ceteris paribus*, be more likely to engage in helping behavior—perhaps because such communality is a useful reaction to the challenges posed by scarce resources (Kraus et al., 2012)—this tendency is offset by the fact that such individuals are more likely to make unfavorable comparisons with similar others, with resultant feelings of resentment and unfairness that are inimical to prosociality. A significant relationship between SSS and prosociality only emerges when criterion-irrelevant variance shared with PRD is removed.

In contrast to the effects of SSS while controlling for PRD, we found no evidence that objective SES indicators (income and education) significantly predict prosociality. This might reflect the difficulty of obtaining sufficiently sensitive indicators of objective SES, with SSS forming a better distillation or “cognitive averaging” (Nielsen, Roos, & Combs, 2015) of relevant information known to the individual but hard to elicit with the methodologies of psychology research. Alternatively, it may be that where one puts oneself on the SSS “ladder” reflects psychological or material circumstances that are not directly related to objective SES indicators at all. In either case, our data urge the importance of considering SSS and objective SES as separate variables rather than interchangeable measures of the same construct.

Relative social status is multifaceted: People compare themselves on multiple dimensions, using multiple frames of references, and combining objective indicators with subjective impressions and affective reactions. Our data illustrate how these complex processes can exert distinct effects on important social behaviors and emphasize the value of simultaneously modeling the effects of distinct components of social status. Objective SES and SSS cannot be treated as equivalent measures of social class, and measuring social class and PRD in isolation risks misjudging the predictive validities of both variables. This approach offers the potential for new insights into previously studied links between social class and outcomes including self-concept, social category formation, and unethical behavior (Kraus et al., 2012) as well as providing a guiding principle for future research into aspects of social functioning for which social class is predicted to be important.

## Supplementary Material

Supplementary material

## Notes

1. This approach is based on the fact that the probability of having an income over a given amount is a power function of that amount and estimates the parameters of this curve from the observed data in lower categories in order to estimate the median of the units falling into the top category. Specifically, if the lower bound of the top category is *X_i_* and this category contains *N_i_* responses, the median for this category is 2^1/*v*^*X_i_*, where $v=\left(\ln\left(N_{i}+N_{i-1}\right)-\ln\left(N_{i}\right)\right)/\left(\ln\left(X_{i}\right)-\ln\left(X_{i-1}\right)\right)$.

2. The same multiple regression analysis adjusting for study (weighted effect coded with four coded vectors) and all possible two-way interactions between study, personal relative deprivation (PRD), subjective socioeconomic status (SSS), and objective socioeconomic status (SES) revealed the same relationships between prosociality and PRD (β = −.217, *p* < .001), SSS (β = −.136, *p* < .001), and objective SES (β = −.013, *p* = .60).
