# Supplementary material for: The Interrelations Between Social Class, Personal Relative Deprivation, and Prosociality
Source: Soc Psychol Personal Sci. 2016 Nov 11;8(6):660–9. doi: 10.1177/1948550616673877 (PMC5641987; doi:10.1177/1948550616673877)
Supplement: Supplementary material [file SPPS673877_suppl_mat.pdf]

Supplementary Materials

1. Instructions for Dictator Game used in Study 2

You are about to play a game...

Please read these instructions carefully:

Imagine yourself in a situation in which your are **given $10**, which you can keep to yourself or give to the next MTurk worker completing this HIT, all or any portion of it.

You will be Player A and the next MTurk worker completing this HIT will be Player B.

The other worker will never know your Worker ID, and you will not know the other player's Worker ID.

You can transfer any amount of the $10 to Player B. You keep the rest of the bonus.

Here are some examples of the game. The examples will be followed by a question.

You must answer the question correctly to ensure your HIT is accepted.

Example 1: Player A transfers $5 to Player B.
Both players get $5 as a bonus.

Example 2: Player A transfers $8 to Player B.
Player A gets $2 bonus, Player B gets $8 bonus.

Example 3: Player A transfers $2 to Player B.
Player A gets $8 bonus, Player B gets $2 bonus.

Please answer the following question to ensure you have understood the game:

1. If Player A (you) transfers $1 to Player B (other worker), how much will Player A get?

☐ ◯ $1
☐ ◯ $9

[page break]

INSTRUCTIONS -- Please read carefully

Now it's time to play the game!

You are Player A. Your partner is Player B (the next MTurk worker).

Imagine you have been allocated a **$10 bonus** for completing this HIT. You can transfer any amount of the $10 bonus to Player B (the next worker).

Please imagine the situation to be as close as possible to a real-life situation.

As an incentive for responding as though you were actually given a $10 bonus, we will be randomly selecting 10 workers from this HIT and paying them a real bonus according to their decisions in this game. An additional 10 workers will receive the amount these workers transferred to Player B. If you are selected, you will receive a bonus according to how much of the $10 you propose to transfer to the next MTurk worker.

Please record your decisions in the boxes below. Please ensure the sum total of the two amounts is **$10**

Player A: I will transfer the following amount to Player B (the next worker):

This means that I will get:

Total

2. Principal Component and Confirmatory Factor Analyses of the Communal Orientation Scale—Studies 4 and 5

Our initial analysis plan was to use the full 14-item Communal Orientation Scale (COS) to investigate the relative importance of personal relative deprivation (PRD) subjective socioeconomic status (SSS), and objective SES in predicting people's communal orientation. However, Consistent with Clark, Ouellette, Powell and Milberg's (1987) results, a Principal Components Analysis of the COS from Study 4 suggested that the scale might tap into two different components rather than one: one's desire to help others (items 2, 3, 4, 5, 6, 8, 9, 10, 12 and 13), and one's desire to receive help from others (items 1, 7, 11 and 14). The PCA extracted 3 components, two with eigenvalues above 2, and one with an eigenvalue above 1. Following inspection of the items and of the scree plot, it was determined that the third component did not illustrate a trend in the items; therefore, only the two components with eigenvalues above 2 are reported below. A reasonable simple structure was obtained, supporting the existence of two components (see Table S1 for factor loadings). The two components accounted for 48.2% of the total variance (Cronbach's $\alpha_{\text{"give help"}}$ = .87, Cronbach's $\alpha_{\text{"receive help"}}$=.62). The two subscales correlated weakly but significantly, *r* = .137, p = .012.

To verify whether the two-factor model is more appropriate, we conducted a CFA of the COS from Study 5. The models were fitted using the *lavaan* package in R using an Unweighted Least Squares estimator. The two-factor model provided a significantly better fit (χ^2 = 1739.25, df = 76, p = NA; CFI = .933; TLI = .920; RMSEA = 0.236; SRMR = .084) than the one-factor model (χ^2 = 4461.09, df = 77, p = NA; CFI = .832; TLI = .791; RMSEA = .381; SRMR = .125), χ^{2diff} = 2721.8, df = 1, *p* < . 001. Standardized factor loadings are shown in Table S1.

Table S1. Factor loadings for the PCA, and standardized estimates for the CFA of the two-factor model, for both Study 4 and Study 5.

COS Items	Study 4 (PCA)		Study 5 (CFA)	
	Factor 1 (give help)	Factor 2 (receive help)	Factor 1 (give help)	Factor 2 (receive help)
**COS1**	.031	**.832**	-	0.650
**COS2**	**.646**	.134	0.605	-
**COS3**	**.647**	-.149	0.677	-
**COS4**	**.602**	-.131	0.707	-
**COS5**	**.554**	.249	0.518	-
**COS6**	**.762**	-.088	0.810	-
**COS7**	.121	**.722**	-	0.677
**COS8**	**.692**	.023	0.660	-
**COS9**	**.779**	-.106	0.748	-
**COS10**	**.729**	-.136	0.704	-
**COS11**	.166	**.287**	-	0.523
**COS12**	**.692**	-.175	0.627	-
**COS13**	**.655**	-.089	0.631	-
**COS14**	.280	**.722**	-	0.823

3. Table S2. *Descriptive Statistics and Inter-correlations for All Measures Used in Studies 4 and 5.*

Measures	*M* (*SD*)	1.	2.	3.	3a.	3b.	4.	4a.	4b.	5.	6.
**Study 4 (*N* = 338)**											
1. PRD	3.00 (0.95)	(.79)									
2. SSS	5.27 (1.68)	-.432*	--								
3. Obj. SES	--	-.179*	.410*	--							
3a. Income (£)	33.9k (27.8k)	-.180*	.325*	.700*	--						
3b. Education	5.01[a] (2.93)	-.069	.249*	.700*	-.021	--					
4. COS	4.99 (0.76)	-.071	-.074	-.138†	-.080	-.113†	(.81)				
4a. Give Help	5.22 (0.93)	-.099	-.092	-.165*	-.132†	-.099	.927*	(.87)			
4b. Want Help	4.42 (1.00)	.042	.017	.017	.094	-.070	.498*	.137†	(.62)		
5. BJW-others	3.35 (1.06)	-.111†	.100	-.091	.004	-.131†	-.167*	-.205*	.033	(.88)	
6. BJW-self	4.47 (1.02)	-.477*	.357*	.105	.114†	.032	-.083	-.085	-.023	.484*	(.89)
Measures	*M* (*SD*)	1.	2.	3.	3a.	3b.	4.	4a.	4b.	5.	
**Study 5 (*N* = 393)**											
1. PRD	3.15 (1.00)	(.81)									
2. SSS	4.94 (1.74)	-.527*	--								
3. Obj. SES	--	-.299*	.533*	--							
3a. Income ($)	51.9k (36.7k)	-.293*	.528*	.794*	--						
3b. Education	2.75 (0.67)	-.181*	.318*	.794*	.262*	--					
4. COS	4.81 (0.88)	-.148*	.036	-.011	.044	-.061	(.84)				
4a. Give Help	5.11 (1.08)	-.238*	.041	.004	.068	-.062	.914*	(.89)			
4b. Want Help	4.04 (1.26)	.146*	.000	-.034	-.039	-.016	.495*	.101†	(.76)		
5. Unfairness	2.88 (1.25)	.759*	-.384*	-.162*	-.209*	-.049	-.136*	-.265*	.236*	(.80)	

Note. PRD = Personal Relative Deprivation; SSS = Subjective Socioeconomic Status; SES = Socioeconomic Status (composite of income and education); COS = Communal Orientation Scale; BJW = Belief in Just World; Alpha reliabilities are presented in parentheses along the diagonals where applicable.
*= $p < .01$, † = $p < .05$; a = Number of years of formal education since the age of 16.

4. Regression Commonality Analyses

We complemented the multiple regression analyses reported in the main text with regression commonality analyses, which were performed using the *yhat* package in R (Nimon, Oswald & Roberts, 2013). Commonality analysis partitions the variance that is explained by all predictors in a multiple regression model into variance that is unique to each predictor (unique effects, or semi-partial correlations squared) and variance that is common to groups of predictors (common effects; Nimon & Reio, 2011). Commonality analysis is well suited to pinpointing predictors that are involved in suppressor situations, with negative values for common effects indicating that a predictor is exerting a suppressor effect. The results of these analyses are summarized in Table S3 (which is the same as Table 4 in the main text but includes the unique and common effects from the commonality analyses). With the exception of SSS in Study 4, the common effects for personal relative deprivation and SSS were always negative. Objective SES, however, showed no consistent suppression effects, given the mix of positive and negative common effects across studies.

Table S3. *Multiple Regression and Commonality Analyses for Studies 1 to 5 and the Collated Data.*

Criterion Predictors	R^2	β_{alone} (*p*) [95% CI]	$\beta_{with\ other\ predictors}$ (*p*) [95% CI]	Unique (sr^2)	Common	Total Suppression Effect	95% BCa CI Suppr. Effect
SVO	.019						
PRD		-.117 (.005) [-.199, -.035]	-.150 (.001) [-.241, -.060]	.019	-.005	.033	-.004, .073
SSS		-.015 (.728) [-.098, .068]	-.086 (.102) [-.189, .017]	.005	-.005	.071	.008, .133
Obj. SES		.001 (.986) [-.082, .084]	.016 (.739) [-.079, .112]	.0002	-.0002	-.016	-.065, .034
AI Community	.093						
PRD		-.176 (<.001) [-.274, -.078]	-.323 (<.001) [-.433, -.213]	.077	-.046	.147	.081, .226
SSS		-.126 (.013) [-.224, -.027]	-.280 (<.001) [-.407, -.154]	.044	-.028	.155	.072, .246
Obj. SES		-.068 (.176) [-.168, .031]	-.015 (.804) [-.131, .101]	.0001	.005	-.054	-.131, .019
Dictator Game	.025						
PRD		-.126 (.003) [-.209, -.042]	-.166 (< .001) [-.258, -.073]	.022	-.007	.040	.002, .082
SSS		-.024 (.578) [-.108, .060]	-.120 (.024) [-.225, -.016]	.009	-.009	.097	.033, .162
Obj. SES		.028 (.521) [-.057, .112]	.048 (.328) [-.049, .146]	.002	-.0009	-.021	-.076, .032
COS-give help (UK)	.052						
PRD		-.099 (.068) [-.206, .007]	-.171 (.004) [-.287, -.055]	.024	-.014	.072	.018, .133
SSS		-.092 (.092) [-.199, .015]	-.103 (.108) [-.228, .023]	.007	.001	.011	-.066, .086
Obj. SES		-.165 (.002) [-.271, .059]	-.154 (.009) [-.269, -.039]	.020	.008	-.012	-.063, .040
COS-give help (US)	.067						
PRD		-.238 (<.001) [-.334, -.141]	-.300 (<.001) [-.413, -.187]	.065	-.009	.062	-.003, .129
SSS		.041 (.419) [-.058, .140]	-.100 (.126) [-.228, .028]	.006	-.004	.141	.050, .234
Obj. SES		.004 (.939) [-.096, .103]	-.033 (.574) [-.146, .081]	.0008	-.0008	.036	-.028, .108
Collated (*N* = 2,233)	.037						
PRD		-.148 (<.001) [-.189, -.107]	-.210 (<.001) [-.257, -.165]	.035	-.013	.062	.041, .085
SSS		-.038 (.072) [-.080, .003]	-.128 (<.001) [-.180,-.077]	.010	-.009	.090	.058, .123
Obj. SES		-.027 (.208) [-.068, .015]	-.013 (.595) [-.061, .034]	.0001	.0006	-.013	-.039, .013

Note. PRD = Personal Relative Deprivation; SSS = Subjective Socioeconomic Status. Obj. SES = composite of income and education; SVO = Social Value Orientation. β_{alone} = zero-order correlation between predictor and criterion. Unique = variance that is uniquely accounted for by a predictor (equivalent to semi-partial correlation squared). Common = the sum of a predictor's common effects. Unique + Common = a predictor's total variance accounted for in a criterion (or r^2). Total suppression effect = the change in β from β_{alone} when modeled with the other two predictors. 95% BCa CI Suppr. Effect = 95% Bias-corrected and accelerated confidence intervals for the total suppression effect.
